# Intensive alveolar recruitment protocol reduces pulmonary complications and intensive care permanence after cardiac surgery

**DOI:** 10.1186/cc12048

**Published:** 2013-03-19

**Authors:** A Leme, L Hajjar, E Nozawa, C Hashizume, J Almeida, J Fukushima, J Auler, R Santiago, R Ianotti, M Amato, E Osawa, M Feltrim, F Galas

**Affiliations:** 1 Heart Institute, São Paulo, Brazil; 2 Faculdade de Medicina da Universidade de São Paulo, Brazil

## Introduction

Cardiac surgical procedures are associated with a high incidence of postoperative complications, increasing costs and mortality. The purpose of this study is to evaluate prospectively the impact of two protective mechanical ventilation strategies, both using low-tidal volume ventilation (6 ml/kg/ibw) after cardiac surgery.

## Methods

We prospectively evaluated 270 patients immediately after cardiac surgery, presenting hypoxemia and PaO_2_/FiO_2 _<250. Patients were randomized to an intensive alveolar recruitment maneuver (ARM) or a moderate ARM strategy. Intensive ARM group: recruitment with an inspiratory pressure amplitude of 15 cmH_2_O and PEEP of 30 cmH_2_O, followed by ventilation with PEEP = 13 cmH_2_O, during 4 hours of protective mechanical ventilation with VT = 6 ml/kg/pbw. Moderate ARM group: recruitment with opening pressures of 20 cmH_2_O in the airways, followed by ventilation with PEEP = 8 cmH_2_O, during 4 hours of protective mechanical ventilation with VT = 6 ml/kg/pbw. The primary outcome was a composite endpoint of severe pulmonary complications in the postoperative period defined as intra-hospital death, need for mechanical ventilation for more than 48 hours after surgery, pulmonary infection or after reintubation within 28 days after randomization. The secondary outcome was the incidence of nonpulmonary complications as postoperative myocardial ischemia, acute renal failure (RIFLE-R), respiratory mechanics and blood gas analysis after ARM, ICU length of stay, hospital length of stay and 30-day mortality.

## Results

The intensive ARM group compared with the moderate ARM group had lower incidence of the primary outcome, mainly due to the reduced rate of pulmonary infection (2.3% vs. 10.1%, *P *= 0.009). Moreover, the intensive ARM group presented higher lung compliance (68 ± 19 vs. 51 ± 17 ml/cmH_2_O, *P <*0.001) and PaO_2_/FiO_2 _ratio (360 ± 68 vs. 240 ± 74, *P <*0.001) after intervention when compared with the moderate group. Also, the intensive ARM presented a lower length of ICU stay (3 days vs. 4 days, *P *= 0.027) than the moderate ARM. There are no differences regarding severe nonpulmonary complications and 30day mortality between groups.

## Conclusion

An intensive ARM strategy reduces postoperative pulmonary complications, reduces hypoxemia, increases lung compliances and decreases the length of ICU stay after cardiac surgery.
